# The use of fluorescence microscopy and image analysis for rapid detection of non-producing revertant cells of Synechocystis sp. PCC6803 and Synechococcus sp. PCC7002

**DOI:** 10.1186/s13104-015-1112-1

**Published:** 2015-04-17

**Authors:** Katja Schulze, Imke Lang, Heike Enke, Diana Grohme, Marcus Frohme

**Affiliations:** Molecular Biotechnology and Functional Genomics, Technical University of Applied Sciences Wildau, Bahnhofstraße 1, 16-2001, D-15745 Wildau, Germany; Algenol Biofuels Germany GmbH, Berlin, Germany

**Keywords:** PCC6803, PCC7002, Genetic instability, Ethanol producer, 3D fluorescence scan, Phycocyanin, Absorption spectra, Fluorescence microscopy, Image analysis

## Abstract

**Background:**

Ethanol production via genetically engineered cyanobacteria is a promising solution for the production of biofuels. Through the introduction of a pyruvate decarboxylase and alcohol dehydrogenase direct ethanol production becomes possible within the cells. However, during cultivation genetic instability can lead to mutations and thus loss of ethanol production. Cells then revert back to the wild type phenotype.

A method for a rapid and simple detection of these non-producing revertant cells in an ethanol producing cell population is an important quality control measure in order to predict genetic stability and the longevity of a producing culture. Several comparable cultivation experiments revealed a difference in the pigmentation for non-producing and producing cells: the accessory pigment phycocyanin (PC) is reduced in case of the ethanol producer, resulting in a yellowish appearance of the culture. Microarray and western blot studies of *Synechocystis* sp. PCC6803 and *Synechococcus* sp. PCC7002 confirmed this PC reduction on the level of RNA and protein.

**Methods:**

Based on these findings we developed a method for fluorescence microscopy in order to distinguish producing and non-producing cells with respect to their pigmentation phenotype. By applying a specific filter set the emitted fluorescence of a producer cell with a reduced PC content appeared orange. The emitted fluorescence of a non-producing cell with a wt pigmentation phenotype was detected in red, and dead cells in green. In an automated process multiple images of each sample were taken and analyzed with a plugin for the image analysis software ImageJ to identify dead (green), non-producing (red) and producing (orange) cells.

**Results:**

The results of the presented validation experiments revealed a good identification with 98 % red cells in the wt sample and 90 % orange cells in the producer sample. The detected wt pigmentation phenotype (red cells) in the producer sample were either not fully induced yet (in 48 h induced cultures) or already reverted to a non-producing cells (in long-term photobioreactor cultivations), emphasizing the sensitivity and resolution of the method.

**Conclusions:**

The fluorescence microscopy method displays a useful technique for a rapid detection of non-producing single cells in an ethanol producing cell population.

## Background

There has been a revamped interest in algae and cyanobacteria biotechnology in recent years, mostly due to the possible applications for biofuel production [1-4]. This study focuses on engineered strains of the model organisms *Synechocystis* sp. PCC6803 and *Synechococcus* sp. PCC7002, which synthesize ethanol from pyruvate through the introduction of pyruvate decarboxylase (PDC) from *Zymomonas mobilis* and additional alcohol dehydrogenase (ADH) from *Synechocystis* sp. 6803. Both genes, contained within a plasmid vector, lead to a branching of fixed carbon towards ethanol production.

Recently the problem of cellular heterogeneity in ethanol producing phototrophic cultures has been recognized and has driven the development of new protocols to study the subpopulations in a photobioreactor (PBR). Even in clonal populations single cells may differ in terms of their genetic composition, physiology and biochemistry [5]. This might have important practical consequences for the productivity and genetic stability of ethanol production in PBRs, as for example it influences the longevity of ethanol production and affects decisions on scale-up and culture management strategies. Internal research at Algenol has shown the mechanisms of the genetic heterogeneity within the ethanologenic vector cassette of an ethanol producing culture to include point mutations, insertions/deletions, and the presence of mobile genetic elements such as transposons. Mostly these genetic instabilities appear in the PDC gene of the ethanologenic cassette and lead to a non-functional PDC expression and therefore a stop in ethanol production.

In ethanol producing cells, fixed carbon is mainly directed into ethanol, leading to a typical phenotype with reduced biomass production, and in case of PCC6803- and PCC7002-based cell lines to a down regulation of the accessory pigment phycocyanin [6]. Changes in the pigmentation of producer cells could be confirmed on RNA and protein level, where a 4-fold reduction in *cpcB,* which encodes the phycocyanin beta subunit, was measured, leading to a severe reduction in the amounts of phycocyanin subunits [6]. As a result of inactivation of the PDC due to the mentioned mutations, the carbon metabolism is switched back to wild type (wt) conditions and the cells recover to a wt pigmentation phenotype.

However in induced cultures the non-producing cells, identified as “revertants”, have a selective advantage in regard to their much faster growth over producing cells and quickly overgrow the ethanol producing subpopulation resulting in loss of productivity. Consequently, the more revertant cells are present in scale-up cultures the earlier a decline in productivity in the reactors can be observed. The quantitative knowledge of reversions allows for pre-emptive measures before loss in ethanol productivity caused by an increasing population of reverted cells becomes crucial.

Today, absorption spectra are used to get an insight into the amount of reverted cells within a culture. Since the phycocyanin content is reduced in ethanol producing cells, an increase of phycocyanin absorption indicates the occurrence of reverting cells. However, when changes become visible within the absorption spectrum, a large amount of reverted cells is already present in the culture, thus resulting in a fast decline of the productivity. Measurements of the ethanol production rate similarly only detect problems when a large number of revertants has already spread through the population. Furthermore, loss in ethanol production can be also a consequence of other limitations, e.g. nutrient limitations, viability and productivity of cells, contaminations, etc.. Therefore the development of a quick and reliable method for quality control of scale-up and ethanol producing cultures that allows the early determination of revertant cells is of high importance.

In this paper we present a simple method for the distinction of the different phenotypes of ethanol producing and “revertant” cells via fluorescence microscopy. The approach is based on a previously developed fluorescence microscopic method [7] which has since also been adapted for high throughput application [8]. It allows a simple and quick viability analysis for cyanobacteria single cells or viability in a mix. The method uses the red fluorescence of chlorophyll to distinguish vital cells from dead cells, which show an unspecific green fluorescence. Through the presented adaptations and further developments of this fluorescence-based cell viability assay, we have developed a protocol to differentiate between the distinct pigmentation phenotypes of producing and non-producing cells of *Synechococcus* and *Synechocystis*.

## Methods

### Culture strains

For the development of a reliable method for rapid detection of non-producing cells two different cyanobacteria strains were used. For both strains we compared wt cultures with the following ethanologenic cultures in order to detect the differences in the pigmentation of a producing and non-producing cell. Details of the two used strains are as followed:

- Synechocystis sp. PCC6803 - Wild type
- ***Synechocystis*sp. PCC6803 - ethanologenic strain #309** including plasmid construct pVZ325-PpetJ-PDC-synADH according to [6] and
- ***Synechococcus*sp. PCC7002 - Wild type**
- ***Synechococcus*sp. PCC7002 - ethanologenic strain TK115** including plasmid construct pGEM-AQ4::smtB-PsmtA_7002_-PDC-PrbcL_6803_-synADH_deg according to [9]

### Cultivation

#### Scale-up procedure

*Synechocystis* sp. PCC6803 and *Synechococcus* sp. PCC7002 wt strain and ethanol producer were grown in 200 ml bubbled reactors at 200 μmol photons*m^−2^*s^−1^ continuous illumination (fluorescent bulbs) and aerated with 0.5% CO_2_-enriched air for five days.

For *Synechocystis* 100 ml of autoclaved seawater BG-11 medium (63 g/l Instant Ocean®, 17.65 mM NaNO_3_, 0.18 mM K_2_HPO_4_, 0.03 mM Citric acid, 0.003 mM EDTA (disodium magnesium), 0.19 mM Na_2_CO_3_, 0.03 mM Ferric ammonium citrate and trace metals: 2.86 mg/l H_3_BO_3_,1.81 mg/l MnCl_2_-4H_2_O, 0.22 mg/l ZnSO_4_-7H_2_O, 0.39 mg/l Na_2_MoO_4_-2H_2_O, 0.08 mg/l CuSO_4_-5H_2_O, 0.05 mg/l Co(NO_3_)_2_-6H_2_O) were used with the addition of 5 × CuSO_4_-5H_2_O (1.6 μM Cu^2+^) for repression, and with 100 mg/ml gentamycin for the ethanologenic culture #309.

For *Synechococcus* the culturing medium was 100 ml autoclaved seawater BG-11 without Zn^2+^, with Vitamin B_12_ (0.004 mg/l), and for the ethanologenic culture TK115 with 200 mg/ml kanamycin.

#### Cultivation for ethanol production

For inoculation of the cultivation experiments the pre-cultures were centrifuged (4500 g, 10 min) and resuspended in induction media.

For *Synechocystis* sp. PCC6803 autoclaved seawater BG-11 without Cu^2+^ was used, since the promoter is induced by copper depletion. For the ethanologenic culture #309 100 mg/ml gentamycin were added to the media.

*Synechococcus* sp. PCC7002 was inoculated in autoclaved seawater BG-11 with 5 μM Zn^2+^ (in this case zinc induces the promoter) and Vitamin B_12_ (0.004 mg/L) and for the ethanologenic culture TK115 with the addition of kanamycin 200 mg/ml.

In order to exclude an impact of zinc addition and copper depletion the wt cultures were grown in the same induction media.

The cultures were cultivated in 250 ml bubbled reactors illuminated from one side with 100 μmol photons*m^−2^*s^−1^ in a temperature controlled chamber allowing a temperature profile with heating to a peak temperature of 38°C for 2 h and cooling to 25°C for 2 h per day. The cultures were aerated with a controlled air flow of 15 ml/min and 5% CO_2_ supply for day and night using a gas mass flow controller. Biomass accumulation (OD_750nm_), ethanol production and absorption spectra were measured daily.

### Absorption spectroscopy and OD_750_ measurement

The spectrum of each culture was measured using the UV-Spectrophotometer UV-1800 (Shimadzu). The spectra of the cultures were recorded between 400 nm and 750 nm. The values were normalized to a relative absorption of 0.45 at the chlorophyll peak at 680 nm to allow a good overview of the ratio between chlorophyll and phycocyanin.

### Three dimensional excitation/emission fluorescence spectroscopy

All cultures were washed with fresh media to eliminate signals from excreted substances and set to an OD_750nm_ of 0.5. All cultures were exited in a range between 350 and 580 nm with a step size of 2 nm. The emission spectrum was recorded in the range of 450–750 nm for every excitation wavelength.

### Sample preparation and fluorescence microscopy

In order to obtain a comprehensive data set of each culture the fluorescence of at least 500–1000 cells was monitored. For this 1 ml of the cultures from the different cultivation systems was transferred to a 1.5 ml reaction tube (OD_750nm_ 1–2) and left with the cap open until microscopy. 30 μl of the cell suspension were transferred on a glass slide and the sample covered with a 24 mm × 24 mm cover glass. The suspension was fixed between the two glasses by light pressure to avoid movement of the cells but without destroying them. The edges of the covers were sealed using nail polish to avoid dry-out of the sample.

For microscopy the automated Olympus CX21 microscope with a 40× Plan Achromat objective was used. For each sample fluorescence images of 20 different positions that were predefined in the automated scanning tool of the Olympus Cell software, were recorded with an exposure time of 800 ms for every sample.

### ImageJ Plugin for automated cell differentiation

The automated differentiation of the cell types was done with an ImageJ plugin that was based on previous works [7]. Irregularities in the illumination of the microscope were corrected with the calculator plus function of ImageJ and an image without sample. To segment all cells from the background the fluorescent images were converted in an RGB-image stack. Afterwards, automated thresholding with the MaxEntropy method was used separately for both, the red and green channel. Both images were combined and the cells were registered using the particle analyzer function of ImageJ. Registered particles smaller than 10 pixel and particles touching the edge of the image were excluded from the analysis to eliminate the influence of artifacts. To enable a classification for each registered particle a histogram of the hue values within the particles was recorded and normalized. Additionally the mean particle brightness was used.

For the differentiation of the three cell types in cultures of *Synechocystis* sp. PCC6803 and *Synechococcus* sp. PCC7002 in each case a neural network was trained separately with an independently created set of training data. For this, a simple feed forward network with one hidden layer based on the Encog java framework [10] was added to the plugin. Training was done with the combination of the resilient propagation algorithm and a genetic algorithm (when the change in the error rate was smaller than 1%) to an error rate of 0%. The trained neural network was used to differentiate the new data into the three classes wt cell without change in the phenotype (red signal), producer cell with changed phenotype (orange signal) and dead cell without photo pigments (green signal).

### Mixing experiment

For the mixing experiment wild type and ethanol producing samples from *Synechocystis* sp. PCC6803 and S*ynechococcus* sp. PCC7002 were each mixed in different ratios.

The *Synechocystis* sp. PCC6803 cultures were obtained after 48 hours of cultivation in induction medium as described under section “*Cultivation for ethanol production”*. The samples were adjusted to a cell density of OD_750nm_ = 1 and mixed in the following percentages of wild type / producer: 0% / 100%, 10% / 90%, 25% / 75%, 50% / 50% and 0% / 100%. For each sample four replicates were measured.

The *Synechococcus* sp. PCC7002 cultures were from running 0.5 l photobioreactor cultivations in induction medium. The samples were adjusted to a cell density of OD_750nm_ = 1 and mixed in the following percentages of wild type / producer: 0% / 100%, 5% / 95%, 10% / 90%, 25% / 75%, 50% / 50% and 0% / 100%. For each sample three replicates were measured.

## Results and discussion

### A prerequisite for the discrimination of non-producer and producer cells

**Figure 1:**
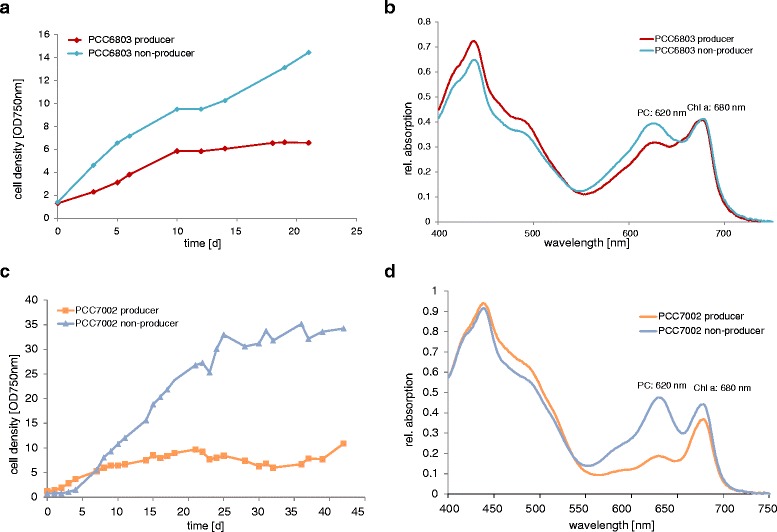
Example of cell growth and changes in the Chlorophyll to Phycocyanin ratio in photobioreactor cultivation experiments. The figure shows the **(a)** cell growth and **(b)** absorption spectrum for induced ethanol producer and wt cells of PCC6803 and the **(c)** cell growth and **(d)** absorption spectrum for induced ethanol producer and wt cells of PCC7002. For both strains the wild type is compared to ethanol producing strains. Due to changes in the carbon partitioning upon ethanol production the induced culture exhibited less cell growth compared to the repressed culture. In addition the amount of phycocyanin (620 nm) was down-regulated in the ethanol producer compared to the non-producing culture which showed a typical wt pigmentation phenotype.

For the development of a method that allows a rapid discrimination of ethanol producing and non-producing cells a distinct and reproducible phenotype is a requisite. This was possible due to fundamental changes in the physiology of the ethanol producing cells in *Synechocystis* PCC6803 and *Synechococcus* PCC7002. Several cultivation experiments at different scale showed that the ethanol producing culture exhibited a yellowish pigmentation phenotype, which was not visible in the non-producing cultures. The measurements of absorption spectra confirmed this observation: the phycocyanin peak at 620 nm was reduced compared to that of the non-producer (see example data in Figure 1).

As mentioned before, microarray and western blot studies of both strains revealed a down regulation of the *cpcB* transcript of the phycocyanin beta-subunit leading to reduced amount of the respective protein [6]. Based on an already established method to determine viable and non-viable cells [7] this distinct pigmentation phenotype of the ethanol producer was used to develop a novel method that allows a quick detection of single cells under the microscope. The development of the presented method was done in several steps described in the following sections:

1. Identification of optimal filter set for fluorescence microscopy
2. Set up of semi-automated fluorescence microscopy
3. Cultivation experiments of wt and ethanol producing strains to generate pigmentation phenotype
4. Mixing studies for a *proof of concept*

### Identification of optimal filter set for fluorescence microscopy

**Figure 2:**
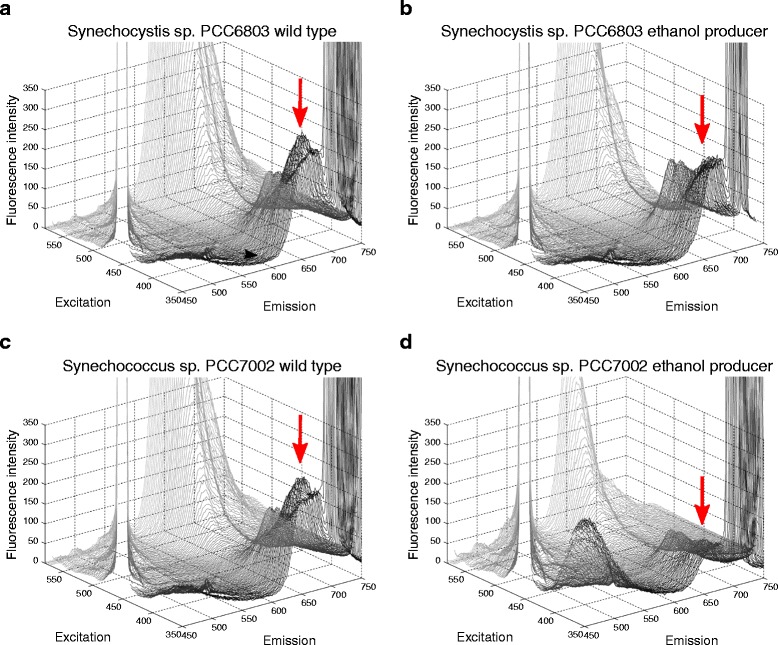
3D-scan of *Synechocystis* PCC6803 wt **(a)** and ethanol producer **(b)** and *Synechococcus* PCC7002 wt **(c)** and ethanol producer **(d)**. The samples were excited with wavelengths between 350 – 680 nm and the fluorescent emission of cells is monitored. Between wt and ethanol producer a clear difference in the emission spectrum was observed at 650 – 680 nm (indicated with arrows).

In order to identify an optimal filter set for fluorescence microscopy the differences found in the pigmentation profile of producing and non-producing cultures (wt) of PCC6803 and PCC7002 were confirmed by applying three dimensional excitation/emission fluorescence spectroscopy. For this, wt and ethanol producing cultures of PCC6803 and PCC7002 taken from ongoing PBR cultivations were excited with wavelengths between 350 – 580 nm and the fluorescent emission of cells was monitored and depicted in a 3D-Scan (Figure 2).

**Figure 3:**
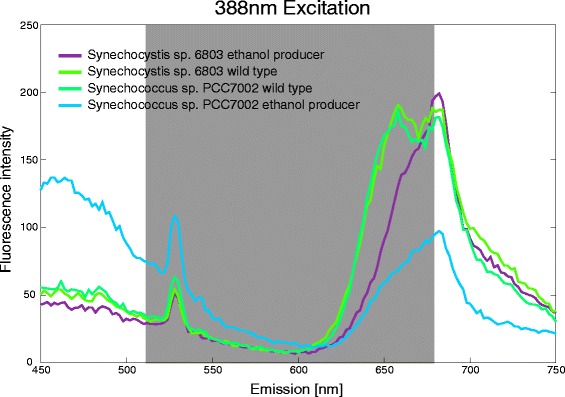
2D emission spectra of wt and ethanologenic cultures of PCC6803 and PCC7002 at 388 nm excitation. The gray marked area indicates the recorded emission wavelength to optimally observe changes in the fluorescence emission.

As observed in previous cultivation experiments, there was an obvious shift in the pigment ratio due to reduced phycocyanin amounts in ethanol producing cultures compared to wt cultures (Figure 1). The ethanologenic strains of PCC6803 and PCC7002 exhibited a smaller peak at 650–680 nm if excited with 388 nm compared to the wt cultures (Figure 2). The difference of wt and ethanol producer was most noticeable if presented in a 2D emission spectrum at the excitation wavelength of 388 nm (Figure 3).

Compared to the wild type both producer strains showed clearly reduced fluorescence signals in the range between 620 and 670 nm, highly likely caused by the reduced PC content in the cells. Additionally an unspecific green signal could be observed for all cultures at around 530 nm. The gray marked area in the 2D emission spectrum (500 – 680 nm, Figure 3) was chosen as the emission wavelength of interest to optimally observe changes in the fluorescence emission caused by a changed ratio between the unspecific green signal and the phycocyanin signal. The optimal filter set thus had an excitation wavelength of 388 nm and an emission of 510 nm long pass. A 680 nm long pass filter (Chroma Technology Corp and Semrock, USA) was used as a cut-off.

### Automated fluorescence microscopy

**Figure 4:**
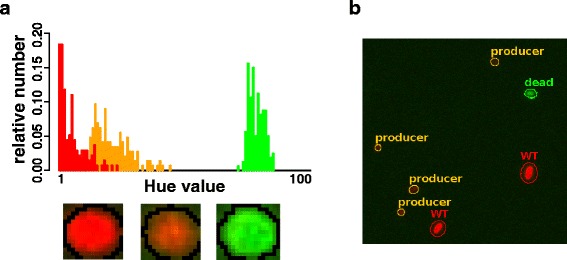
Examples for the differentiation of different cell stages. **(a)** Fluorescence signals of the three different cell stages with a color histogram of the corresponding cell (the area used for the histogram is surrounded with a black line) and **(b)** Example of the automated recognition of the different cell stages of PCC6803 using the ImageJ software. Cells that appeared red represented the wt pigmentation phenotype and cells that appeared orange represented the pigmentation phenotype of ethanol producing cultures. Cells in green were dead.

The automated fluorescence microscopy was implemented on an Olympus CX21 microscope to recognize non-producing, ethanol producing and dead cells in a sample; based on the previously demonstrated reduced phycocyanin content exhibited by ethanol producing cells compared to non-producing cells. Due to the chosen filter set (excitation: 370 – 410 nm; beam splitter: 495 nm; emission: 510 nm long-pass and cut off 680 nm), the ethanol producing cells appeared orange and less bright (since the red signal and the green signal showed approximately the same intensity) and wt / non-producing cells red (since the red signal had a much higher intensity) under the microscope (Figure 4a). Dead cells were recognized with green fluorescence since no photo pigments were present in the cells [7]. For the automated recognition of the different cell types a plugin was written for the free software ImageJ [11]. After an initial training of the used classifier with separate data a color histogram was generated from each cell and classified in dead (green), producer (orange) and wt or non-producing revertant cells (red) (Figure 4b).

### Cultivation experiments of wt and ethanol producing strains to generate pigmentation phenotype and confirmation of the microscopic method

#### Biomass and ethanol productivity of wt and ethanologenic cultures of PCC6803 and PCC7002

**Figure 5:**
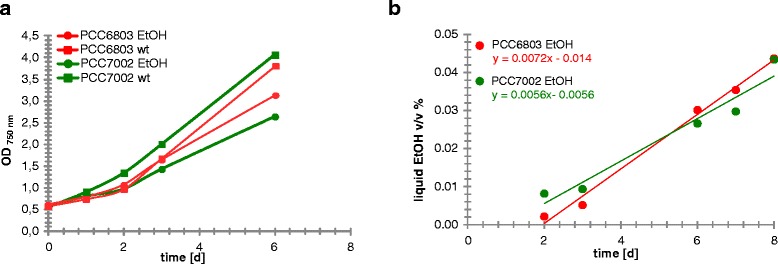
Cell growth and ethanol production. **(a)** Cell growth of wt and ethanol producing cultures of *Synechocystis* PCC6803 (red) and *Synechococcus* PCC7002 (green) **(b)** Ethanol production [v/v%] and respective daily rates of PCC6803 (red) and PCC7002 ethanol producer (green).

The cultures were grown for one week in bubbled reactors and monitored for cell growth, ethanol production and absorption spectra, while the fluorescence signals of the cultures under the microscope were compared in parallel. After a lag phase in productivity of two days, wt and ethanol producing cultures of PCC6803 and PCC7002 started to accumulate biomass and produce ethanol (Figure 5). The difference in biomass accumulation between wt and ethanol producer was more pronounced in PCC7002 than in PCC6803 (Figure 5a). This could be explained by the different induction systems for ethanol production. As mentioned before PCC7002 TK115 ethanol production was induced by the addition of zinc ions, the ethanol producer of PCC6803 was induced by copper depletion. Residual copper concentration in the cultures would impact the length of transition to a fully induced promoter system. Therefore the cells of PCC6803 would fix more carbon into biomass than into ethanol. If the cultures grew for a longer period the difference between wt and ethanol producer would have been more obvious, as observed in previous cultivations. The ethanol productivity was higher in PCC6803 with 0.0072 v/v% *d^−1^ than in PCC7002 with 0.0056 v/v%*d^−1^ (Figure 5b).

#### Pigmentation and microscopical analysis of wt and ethanol producing cultures of PCC6803 and PCC7002

**Figure 6:**
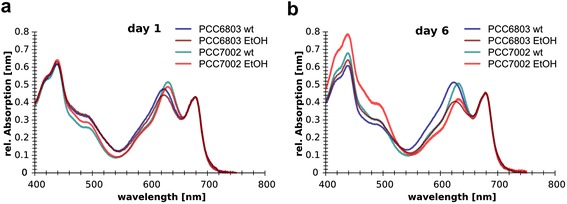
Absorption spectra of PCC6803 and PCC7002 **(a)** before induction and **(b)** on day 6 after induction. The pigmentation of both ethanol producers (EtOH) changed with cultivation length and exhibited a shift in the PC / Chl ratio (620 nm / 680 nm) compared to the wt. The addition of ZnSO_4_ as inducer for PCC7002 had no impact on the pigmentation phenotype as shown for the wt culture spiked with 5 μM ZnSO_4_.

The absorption spectrum of each culture was monitored before and after induction and during the length of the experiment. The absorption spectrum of wt and ethanol producing cultures was recorded daily until changes in the pigment ratios (PC / Chl) were detected. In parallel the samples were examined under a fluorescence microscope using the described filter set. For both ethanologenic producer strains the amount of phycocyanin progressively declined compared to the wt culture from day 1 to 6 resulting in the observed shift in PC / Chl ratio due to the reduced PC content. Whereas the pigment ratio of PC to Chl remained constant in the wt cultures (Figure 6).

**Figure 7:**
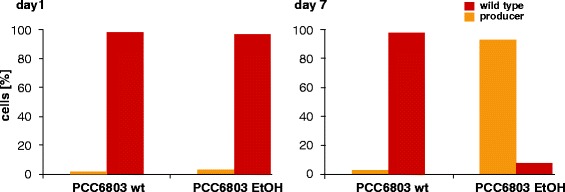
Amount of recognized wt (red) and ethanol producing cells (orange) of the *Synechocystis* sp. PCC6803 culture. Values represent mean of duplicates.

In parallel to the monitoring of the absorption spectra the cell populations were analyzed via automated fluorescence microscopy as described above. As expected, one day after inoculation the majority of cells in wt and ethanol producing cultures of PCC6803 were assigned to the wt pigmentation phenotype (Figure 7). A small minority of cells appeared orange and was therefore recognized as ethanol producing phenotype. The scale-up cultures were maintained in repression medium (which does not allow the expression of the promoter) to allow a faster scale up of the cultures, since the ethanol production slows the growth rate. Thus the ethanologenic phenotype is not expected at this stage. One explanation for the detected orange cells could be the light and medium induced stress due to the changes in cultivation conditions upon inoculation. In particular the increased light availability after inoculation influences the chlorophyll and phycocyanin content of the cells. After completion of the scale up, cultures are diluted into induction medium to start the production of ethanol. Seven days after inoculation the wt culture was still recognized as wt whereas the majority of ethanol producing cells were assigned to the respective pigmentation phenotype (Figure 7). Very similar results were obtained with the PCC7002 cultures (data not shown). The results of the fluorescence microscopy method were in good agreement with the measured absorption spectra and the protocol could be used to distinguish between cells that produce and those that do not produce ethanol.

### Mixing study as proof of concept

As a validation of the method we prepared mixed samples of wt and ethanol producing cells of PCC6803 and PCC7002. For PCC6803, the wt and ethanol producing samples were derived from induced cultures after 48 hours of cultivation in bubbled reactors. In case of *Synechococcus* sp. PCC7002 the samples derived from running lab-scale cultivation experiments in 0.5 l photobioreactors. Both showed ethanol production and the previously described difference in the pigmentation phenotype. Before mixing, both cultures were adjusted to a cell density of OD_750nm_ = 1 and then mixed to aimed percentages of wt cells (see Methods). As reference pure samples of wt and ethanol producing cells were measured as well.

**Figure 8:**
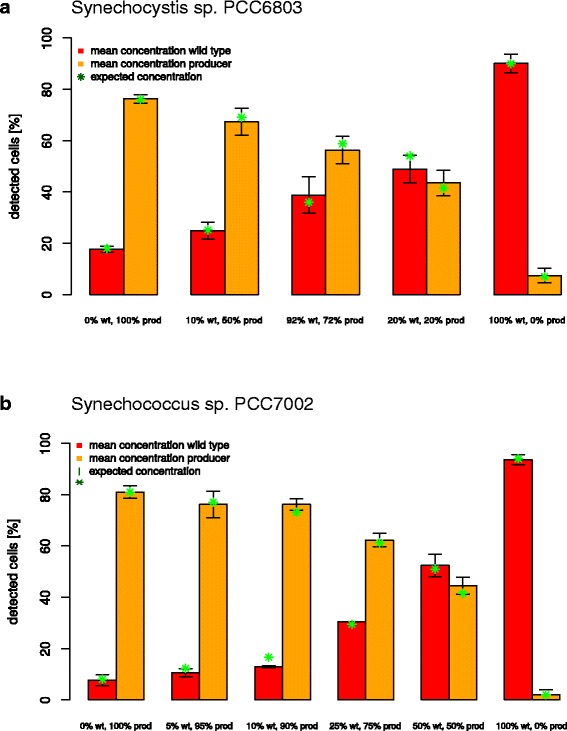
Amount of recognized wt (red) and ethanol producing cells (orange) in different mixtures of **(a)** PCC6803 and **(b)** PCC7002 wt and ethanol producing cells. Points in green indicate the expected amount of wt and ethanol producing cells. For PCC6803 the cells derived from Erlenmeyer cultures that were induced for 48 h. For PCC7002 the cells derived from running 0.5 l photobioreactor experiments in induction medium. For both strains wt and ethanol producing cultures exhibited different pigmentation phenotypes due to the absorption spectra. The values represent a mean of three replicates.

The analysis showed that the wt and ethanol producing samples of both strains contained dead cells in different amounts (PCC6803: wt – 2.5%, ethanol producer – 6%; PCC7002: wt – 4.4%, ethanol producer – 11.3%). Furthermore, the analysis of the microscopic pictures revealed that 90.1% cells of the PCC6803 wt culture were recognized as wt cells but a small percentage of 7.4% showed a pigmentation phenotype assigned to ethanol producing cells (Figure 8a). With 93.6 % of detected wt cells and 2% of ethanol producing cells similar results were observed for PCC7002 (Figure 8b).

The cells in the wt strains that were misidentified as producers due to their orange emission, exhibited a changed phenotype not associated to the ethanol production. Probably these were cells in the process of dying and thus also showed a reduced content of photo pigments. With the method used, it is not possible to distinguish between these different causes for the changed phenotype. However, this is not a drawback for its practical application, as the focus of the method is the detection healthy revertant (wt) cells within a producer culture. Only these cells have the potential to overgrow the rest of the population due to their increased growth rate.

In case of the ethanol producing culture of PCC6803 and PCC7002, 76.3% and 81.0% cells were recognized as ethanol producing cells, but a portion of 17.7% and 7.7% still exhibited the pigmentation phenotype of wt cells (Figure 8). An explanation of wt cell detection in the ethanol producing population could be the presence of non-producing cells which either were not fully induced yet or exhibited a mutation in the PDC gene cassette resulting in loss of ethanol productivity. In both cases the PC / Chl ratio would be identical to that of wt cells. Here has to be noted that it is hardly possible to maintain an “ideal” 100% producer culture since it is never possible to induce all cells. Additionally the reversion of the cells can occur in any state of the cultivation process.

As a consequence of dead cells and the not 100% pure start samples the results of the mixing experiments of wt and ethanol producing cells were expected to deviate from the calculated optimal percentages. Therefore, based on the results for the pure samples, the expected percentages in the mixed cultures were adapted and showed a good correlation to the results that were obtained in the experiment (Figure 8).

## Conclusion

Autofluorescence displays a useful parameter to investigate phototrophic single cells, for example for cell viability [7], but it also represents a very sensitive parameter in regards to changes in chlorophyll and phycobillisome content upon stress. In the present method we used the difference in the pigmentation phenotype to distinguish between non-producing and ethanol producing cells in a PBR on a single cell level.

The reduction of phycocyanin in ethanol producing cells of *Synechocystis* PCC6803 and *Synechococcus* PCC7002 could be evidently attributed to the cell’s response on ethanol production and not on ethanol itself. In both ethanol producing strains PC reduction has been observed on both levels of gene expression (transcript level and protein level). Those reductions resulted in different absorption spectra of ethanol producing cells versus non-producing wt and revertant cells and consequently also in different emission spectra which were visualized by using a specific filter set for fluorescence microscopy. Compared to the previously used method of measuring an absorption spectrum of the whole culture, the newly established analysis with the microscopical assay enables conclusions on the cell level, thus allowing a more precise and earlier detection of revertant cells. In addition the method allowed for the detection of dead or damaged cells, which show an unspecific green fluorescence. We performed cultivation experiments to confirm the applicability of this method as a simple and reliable quality control check for ethanol producing cultures of *Synechocystis* PCC6803 and *Synechococcus* PCC7002.

The assay might be applicable to other cyanobacterial expression systems as long as the cell pigmentation is affected, which is often expected as the redirection of nutrient flow causes starvation like conditions within the cell. Additionally the use of this new method within a FACS could enable a fast sorting of cultures before inoculation of production reactors and thus enable higher production rates.
